# The use of pre-operative computed tomography in the assessment of the acute abdomen

**DOI:** 10.1308/003588412X13373405386132

**Published:** 2012-09

**Authors:** Zubair Khanzada, Nick Lagatolla

**Affiliations:** Dorset County Hospital,Dorchester

## Abstract

**Weir-McCall J, Shaw A, Arya A, Knight A, Howlett DC** The use of pre-operative computed tomography in the assessment of the acute abdomen. *Ann R Coll Surg Engl* 2012; **94**: 102–107 doi: 10.1308/003588412X13171221501663

The authors have presented a study correlating the reports of abdominal computed tomography (CT) in the acute abdomen with findings at laparotomy. The retrospective diagnostic ‘accuracy’ of CT scan reporting rises from 78% when reported by a registrar to 83% when additionally assessed by a consultant and finally to 93% after a further consultant review. In their conclusions, the authors state that this represents ‘a high degree of accuracy’. This mirrors our experience: we also feel that the correct diagnosis is missed by CT scan in approximately one to two patients in every ten presenting with an acute abdomen requiring surgery.

However, we are concerned that the authors have totally ignored the impact that a full clinical assessment would have on correct diagnosis. We feel strongly that radiological techniques are an adjunct, albeit important, to clinical history taking and examination in the formulation of a working diagnosis. In this way, the majority of patients may be correctly diagnosed pre-operatively, which ought to be the minimum standard.

